# Declining Influenza Vaccination Coverage among Nurses, Hong Kong, 2006–2012

**DOI:** 10.3201/eid1910.130195

**Published:** 2013-10

**Authors:** Shui Shan Lee, Ngai Sze Wong, Sing Lee

**Affiliations:** The Chinese University of Hong Kong, Hong Kong Special Administrative Region, People’s Republic of China

**Keywords:** seasonal influenza, vaccination, health care workers, influenza, viruses, nurses, Hong Kong

## Abstract

Seasonal influenza vaccination of nurses in Hong Kong fell from 57% in 2005 to 24% in 2012, paralleling concern for adverse reactions associated with vaccination. Decreased acceptance of vaccination was most prominent among nurses who had less work experience and more frequent contact with patients.

Despite the moderate effectiveness of influenza vaccination against seasonal infections (*1*), vaccination remains a key prevention strategy for enhancing population preparedness. Vaccination of health care workers serves a dual purpose: self-protection and reduction of transmission in health care settings. Worldwide, vaccination coverage among health care workers is extremely variable: 20%–40% in western Europe (*2*), 16%–60% in Australia (*3*), 63.5% in the United States (*4*), and 30% in Hong Kong (*5*). The main problems with vaccination studies are their incomparability and the changing patterns over time. To determine patterns in Hong Kong, we investigated an 8-year trend of vaccination coverage among nurses.

## The Study

In Hong Kong, free yearly influenza vaccination has been offered to all health care workers in the public service since 2003, in the aftermath of severe acute respiratory syndrome. In collaboration with 3 local nurses associations, we administered postal surveys every 1–2 years, focusing on vaccination of nurses and the reasons for their acceptance or decline. The surveys were conducted in 2006, 2007, 2009, 2011, and 2012; during each of these 5 years, 2,500–3,000 questionnaires in the Chinese language were delivered by postal service. Coverage of the surveys ranged from 26% to 38% (Table 1). The surveys included core questions about whether respondents had been vaccinated before the past winter season and whether they planned to be vaccinated in the next year. An incentive, in the form of a book or cash coupon for 25 Hong Kong dollars (1 US$ = 7.8 Hong Kong dollars), was offered to respondents who returned a completed survey form before the end of a 1-month period. Approval of the Survey and Behavioral Research Ethics Committee of the Chinese University of Hong Kong was obtained. Snapshot results of the survey in 2006, 2009, and 2011 have been published (*6*–*8*).

**Table 1:** General characteristics of questionnaire about influenza vaccination and nurses’ responses

Characteristic	Date of survey

2006 Sep–Oct	2007 Oct–Nov	2009 July–Aug	2011 Mar–Apr	2012 Feb–Mar
General					
No. questionnaires delivered	2,825	2,929	2,929	2,494	2,492
Response rate, %	38.2	26.9	27.7	28.4	26.6
Total no. respondents	903	739	779	477	651
Male, %	11.7	14.1	14.2	17.6	15.1
Reasons for getting vaccinated, no. respondents*	510	339	306	127	161
Self-protection, %	74	88	87	94	91
Protecting others, %	32	58	53	71	67
Work requirement, %	38	55	62	39	33
Reasons for not getting vaccinated,* no. respondents	377	397	451	350	490
Concern about side effects, %	42	55	62	70	56
Ineffectiveness for self-protection, %	50	57*	51	45	50
Ineffectiveness for protecting others, %	9	22	14	13	23
Lack of work requirement, %	7	9	6	6	5

*A multiple-choice list was provided for respondents. Only common reasons specifically chosen by respondents in each survey are included in the table, in order of their preferences. Less than 5% chose “other” as their reason for getting vaccinated, whereas ≈20% chose “other” as their reason for not getting vaccinated.

In Hong Kong, influenza vaccination is administered from October through February. In each of the years from 2005 through 2012, the vaccination rate for nurses before the previous winter season was captured. In 2007 and 2012, the percentage of nurses intending to be vaccinated was collected because surveys had not been organized in time to record the actual rate in those years. In 2005, the vaccination rate was 56.8%. It ranged from 50% to 60% irrespective of age, sex, professional rank (registered nurse or enrolled nurse), amount of work experience, and intensity of patient contact (always, sometimes, seldom, or never) (Table 2). A steady decline in vaccination of nurses was observed, more prominently after 2009; the prediction for 2012 was for vaccination of <25% of nurses (Figure). The rates have diversified between subgroups of nurses, despite similarity by sex and professional rank (Table 2). The largest decline in vaccination occurred among nurses with less work experience. Whereas 51% of those with <10 years’ experience were vaccinated in 2005, only 10% were vaccinated in 2012. Surprisingly, nurses always in contact with patients were less inclined to be vaccinated; during this period, the rate of vaccination coverage fell from 54% to 23%. Similarly, vaccination rates among those seldom or never in contact with patients also declined, from ≈60% in 2005 to ≈25% in 2010. Thereafter, the vaccination rate increased to ≈40% in those seldom in contact with patients but continued to fall to ≈20% among nurses not needing to work with patients.

**Table 2:** Influenza vaccination rates and characteristics of nurses, Hong Kong

Characteristic	Year	

2005	2006	2007*	2008	2009†	2010	2011	2012*
Total sample size	903	739	739	779	477	477	651	639
Winter influenza season	Mar–Jul**‡**	Feb–Apr	Feb–May	Feb–Mar	Jan–Mar	Mar–-May	Jan–Mar	Jan-Mar
Predominant influenza virus strain	A(H3N2)	A(H1N1)	A(H3N2)	A(H1N1)	A(H1N1)	B	A(H1N1)2009	B
Overall vaccination rate, % (95% CI)	56.8 (53.6–60.0)	46.0 (42.4–49.6)	41 (37.6–44.7)	39.8 (36.3–43.2)	39.8 (35.4–44.2)	26.6 (22.6–30.6)	24.7 (21.4–28.1)	24.4 (21.1–27.8)
Vaccination rate by subgroup								
Sex, no. (%)								
M	106 (58)	104 (44)	104 (42)	111 (44)	84 (39)	84 (33)	98 (31)	95 (29)
F	797 (57)	635 (46)	635 (41)	668 (39)	393 (40)	393 (25)	553 (24)	544 (24)
Age, y, no. ( %)								
≤35	225 (52)	104 (38)	104 (30)	85 (44)	22 (36)	22 (23)	88 (15)	86 (16)
36–45	422 (59)	363 (45)	363 (40)	318 (36)	138 (32)	138 (28)	159 (25)	155 (26)
>45	256 (57)	272 (50)	272 (47)	376 (42)	317 (44)	317 (26)	404 (27)	398 (25)
Professional rank, no. (%)								
Registered nurse§	670 (57)	558 (46)	558 (41)	636 (40)	397 (40)	397 (27)	537 (24)	527 (24)
Enrolled nurse#	203 (55)	163 (44)	163 (39)	124 (41)	77 (38)	77 (22)	96 (27)	94 (24)
Working experience, no. (%)								
<10 y	146 (51)	57 (42)	57 (35)	71 (44)	21 (33)	21 (19)	80 (14)	78 (10)
11–20 y	373 (58)	275 (40)	27 (35)	240 (37)	103 (30)	103 (22)	151 (22)	147 (23)
>20 y	356 (59)	385 (51)	385 (46)	452 (41)	353 (43)	353 (28)	416 (28)	410 (28)
Patient contact, no. (%)								
Always	679 (54)	546 (42)	546 (37)	521 (39)	311 (37)	311 (26)	447 (23)	442 (23)
Sometimes	79 (59)	62 (60)	62 (50)	76 (37)	41 (44)	41 (34)	64 (33)	62 (27)
Seldom	79 (68)	71 (55)	71 (55)	83 (49)	50 (44)	50 (24)	51 (39)	47 (45)
Never	59 (66)	57 (53)	57 (49)	93 (40)	7 (46)	72 (26)	87 (17)	86 (17)

*Planned vaccination as indicated by nurses in the preceding year. †Vaccination history based on nurses’ recall the following year. **‡**Winter and summer seasons merged, giving an extended period of influenza activities. §Completed 3 yr of training in a recognized school and holds a degree/diploma in nursing or equivalent. #Completed 2 yr of training in a recognized training school under a hospital-based program.

**Figure:**
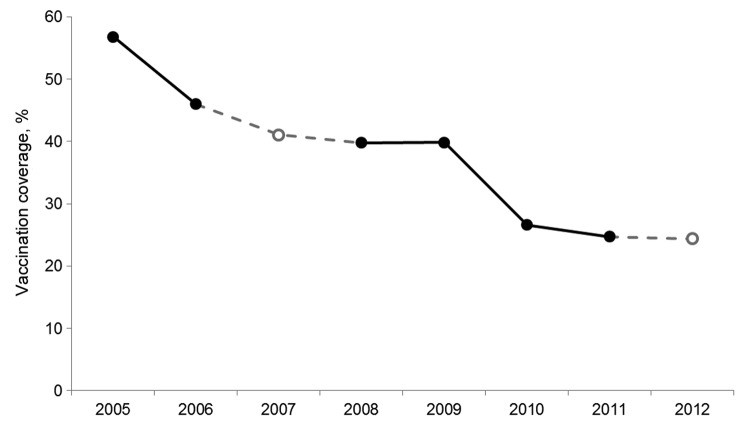
Rates of influenza vaccination among nurses, before the respective winter seasons, Hong Kong, 2005–2012. Closed circles indicate data based on nurses’ recall a year later; open circles indicate data based on nurses’ plans to get vaccinated the next year.

Overall, self-protection was the main reason for receiving seasonal influenza vaccination, increasing from 74% in 2006 to >90% in 2011 and 2012 (Table 1). The proportion of nurses who indicated that “protecting others” was their reason for vaccination almost doubled since 2006, reflecting nurses’ improved understanding of infection control principles. Lack of a work requirement for vaccination was not a main reason for not getting vaccinated because vaccination is not mandatory in Hong Kong. For nurses who refused to be vaccinated, the main reason was “concern for side effects,” as indicated by 42% in 2006, 70% in 2011, and 56% in 2012. A smaller proportion of respondents indicated refusal to be vaccinated because of “ineffectiveness for self-protection” and “ineffectiveness for protecting others.”

Apparently, attitudes of nurses could be influenced by prevailing epidemic situations locally and internationally. Elsewhere, increased vaccination during the 2009 influenza A(H1N1) pandemic has been reported (*9*), a phenomenon similar to that in Hong Kong after the 2003 outbreak of severe acute respiratory syndrome (*6*). Our serial surveys indicated a peak rate of 57% in 2005, which fell to 46% in the next year. For some experienced nurses (>20 years’ experience, ages >45 years), vaccination coverage rate was ≈50% in 2006, when outbreaks of infectious diseases, notably avian influenza, became evident in neighboring mainland China (*10*). The gradual decline of vaccination coverage paralleled the relatively quiet years; it followed the usual bimodal seasonal peaks until influenza A(H1N1)pdm09 virus emerged. Before this new epidemic, the vaccination rate was 40%, comparable to the rate determined by another study of health care workers in Hong Kong (*11*). In retrospect, by the time seasonal vaccination was offered toward the end of 2009, the first wave of A(H1N1)pdm09 infection had already subsided. Vaccination coverage in the next year fell to 30%. In addition to the seasonal vaccine, a new monovalent vaccine against A(H1N1)pdm09 virus was introduced at the same year end. The new vaccine was accepted by only 14.2% of the nurses, as indicated by our 2011 survey results (*8*). The association of A(H1N1)pdm2009 vaccine with Guillain-Barré syndrome was a deterrent to vaccination, despite the minimal increased risk (1 case/1 million vaccinees) (*12*), especially in light of the mild nature of influenza disease caused by A(H1N1)pdm09 virus. The incorporation of A(H1N1)pdm09 virus in the trivalent vaccine at the end of 2010 could be one reason for the precipitous fall in vaccination coverage (*8*).

Our study had some limitations. The small sample size and focus on nurses, most of whom were in the public service, imply that generalization of the results to all health care workers would need to be made with caution. Also, repeated administration of the same survey might have led some respondents to choose a desirable rather than an appropriate answer. In addition, although the profile of respondents has remained similar, minor changes in characteristics might have occurred, making interpretation of longitudinal patterns difficult.

## Conclusions

Influenza vaccination among health care workers is a complex issue. Our results suggest that whereas self-protection was the main reason for choosing seasonal influenza vaccination, concern about side effects was the main reason for refusing to be vaccinated. A balance of these 2 factors varies with time and is associated with the epidemic condition perceived by the nurses in this study. To enhance societal preparedness, influenza vaccination coverage among health care workers should be maintained at a high level. Those who are at highest risk for virus transmission, i.e., those on the front line and in frequent contact with patients, should be the primary focus of vaccination campaigns. The temporal trend of health care workers’ acceptance of seasonal influenza vaccination was evidently not uniform. For future vaccination strategies, measures for improving vaccination coverage should be tailored to the needs of subcategories of health care workers, including nurses, as defined by their potential for virus transmission.
